# Liver abscess due to fish bone ingestion

**DOI:** 10.11604/pamj.2019.32.26.17822

**Published:** 2019-01-16

**Authors:** Rodolfo Mendes Queiroz, Fred Bernardes Filho

**Affiliations:** 1 Department of Radiology and Imaging, Santa Casa da Misericórdia of Avaré, Avaré, São Paulo, Brazil; 2 Centromed Diagnóstico por Imagem, Avaré, São Paulo, Brazil; 3 Dermatology Division, Department of Medical Clinics, Ribeirão Preto Medical School, University of São Paulo, Ribeirão Preto, Brazil

**Keywords:** Liver abscess, fish bone ingestion, peptic ulcer

## Image in medicine

A 50-year-old previously healthy male presented with epigastric pain for 10 days and fever in the last three days. Laboratory testing was remarkable for elevated white blood cells at 19,580/mm^3^ and C-reactive protein of 83.9 mg/dl. Abdomen computed tomography showed a small linear structure with calcium density, transfixing the wall of the gastric antrum and penetrating the left hepatic lobe; an oval massive hypodensity was observed in the surrounding liver parenchyma (A); pneumoperitoneum, free intra-abdominal fluid or vesicular changes were not observed. The hypothesis of gastric perforation due to ingestion of a foreign body with abscess formation was raised. In a new clinical interview, the patient reported a routine habit of fish consumption. Abdominal surgical approach was performed which confirmed the presence of a fish bone and the hepatic collection with purulent fluid (B). After seven days of surgery and treatment with intravenous ceftriaxone and metronidazole, the patient evolved with clinical and laboratory improvement and was discharged. Gastrointestinal perforation by an ingested fish bone resulting in hepatic abscess is very rare. In these cases, the site of perforation is usually in the stomach or duodenum with the abscess most commonly developing in the left hepatic lobe. The classic indicators of hepatic abscess, such as fever with chills, abdominal pain and jaundice are present in only a small number of patients. Among several causative objects that can perforate the gastrointestinal tract are included toothpicks, sewing needles, hairpins, wire, fish bones, chicken bones and dental plates.

**Figure 1:**
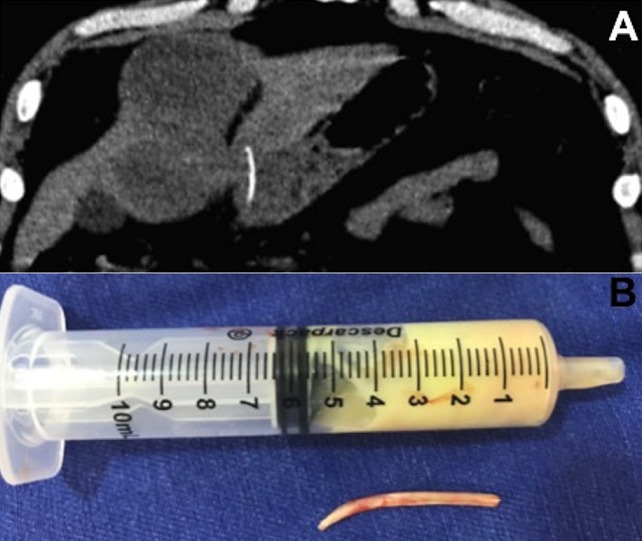
A) abdomen computed tomography showing a small linear structure with calcium density, transfixing the wall of the gastric antrum and penetrating the left hepatic lobe and an oval massive hypodensity; B) purulent fluid

